# Sequencing-informed, patient-specific droplet digital PCR assays for circulating tumor DNA monitoring in recurrent or metastatic head and neck cancer: a pilot study

**DOI:** 10.3389/fonc.2026.1984504

**Published:** 2026-10-30

**Authors:** Luca Leonardo Amodio, Lea Grossmann, Stefanie Gächter, Marta Nowak, Myriam Schaufelberger, Christina Mennicke, Sepehr Rahmani-Khajouei, Martin Zoche, Holger Moch, Achim Weber, Ralph Fritsch, Tomas Brezina, Saskia Hussung

**Affiliations:** 1 Department of Medical Oncology and Hematology, University Hospital Zurich, Zurich, Switzerland; 2 Institute of Pathology and Molecular Pathology, University Hospital Zurich, Zurich, Switzerland; 3 University of Zurich, Zurich, Switzerland; 4 Institute of Molecular Cancer Research (IMCR), University of Zurich, Zurich, Switzerland; 5 Comprehensive Cancer Center Zurich, Zurich, Switzerland

**Keywords:** circulating tumor DNA (ctDNA), droplet digital PCR, liquid biopsy, personalized treatment, precision oncology, recurrent/metastatic head and neck cancer

## Abstract

**Background:**

In recurrent or metastatic head and neck squamous cell carcinoma (R/M HNSCC), early identification of treatment response remains challenging because imaging may be delayed or confounded by treatment-related changes and atypical response patterns under immunotherapy. Serial circulating tumor DNA (ctDNA) analysis may provide a minimally invasive measure of tumor dynamics and complement conventional imaging. We therefore investigated the feasibility and clinical relevance of patient-specific droplet digital PCR (ddPCR)-based ctDNA monitoring.

**Methods:**

Nine patients with R/M HNSCC underwent tumor genomic profiling using FoundationOne^®^ CDx as part of routine clinical care. Patient-specific single-nucleotide variants, including pathogenic or likely pathogenic variants and technically suitable variants of uncertain significance, were selected for ddPCR assay development. Plasma samples were collected before and/or during systemic treatment, and ctDNA kinetics were assessed in relation to radiologic response, progression-free survival (PFS), and overall survival (OS).

**Results:**

Sixteen single-target ddPCR assays were successfully established and combined into ten patient-specific single- or multi-target assay configurations. A total of 21 plasma samples were analyzed, and technically valid measurements were obtained for all samples. Pretreatment plasma was available for seven patients, of whom three (42.9%) had detectable ctDNA. Across all time points, ctDNA was detected at least once in six of nine patients (66.7%). Among the six patients with serial sampling, ctDNA kinetics showed descriptive agreement with radiologic assessment in five (83.3%). The single patient with increasing ctDNA developed progressive disease, whereas decreasing or stable ctDNA was accompanied by partial response or stable disease in four patients; one patient showed a mixed radiologic response. In an exploratory descriptive comparison, the single patient with increasing ctDNA had shorter observed PFS and OS than the five patients with decreasing or stable ctDNA levels. Given the group size of one patient, this observation is hypothesis-generating and does not establish a prognostic association.

**Conclusions:**

Patient-specific ddPCR-based ctDNA monitoring was technically feasible and showed agreement with radiologic assessment in most evaluable patients. These proof-of-concept findings support prospective evaluation of patient-specific ddPCR as a focused approach for longitudinal monitoring in R/M HNSCC; its comparative clinical utility and cost-effectiveness remain to be established.

## 1 Introduction

Head and neck squamous cell carcinoma (HNSCC) ranks among the most common malignancies worldwide (1, 2). Despite advances in multimodal treatment with curative intent, a substantial proportion of patients develop locoregional recurrence or distant metastases (3). In recurrent and/or metastatic (R/M) HNSCC, immune checkpoint inhibitors (ICI) targeting the programmed cell death protein 1/programmed death-ligand 1 (PD-1/PD-L1) axis have become an established component of systemic therapy (4–8). However, durable clinical benefit is limited to a subset of patients, and reliable biomarkers for the early assessment of treatment response remain lacking. Treatment response is currently assessed primarily by computed tomography (CT), magnetic resonance imaging (MRI) and 18F-FDG positron emission tomography (PET/CT) (9). Although these imaging modalities are indispensable in clinical practice, their interpretation may be complicated by treatment-related inflammation, fibrosis, and other post-therapeutic tissue changes. In addition, their sensitivity may be insufficient for detecting small-volume or micrometastatic disease. In patients receiving immunotherapy, assessment is further complicated by atypical response patterns, including pseudoprogression, which may be misclassified as progressive disease using conventional Response Evaluation Criteria in Solid Tumors (RECIST) (10–12). Biomarkers that complement imaging and provide an earlier and more dynamic measure of disease activity are therefore needed.

Circulating tumor DNA (ctDNA), the tumor-derived fraction of cell-free DNA shed into the circulation, has emerged as a minimally invasive “liquid biopsy” with potential applications in molecular profiling, detection of minimal residual disease (MRD), and longitudinal monitoring of treatment response (13–21). Because blood sampling is minimally invasive and can be repeated throughout treatment, ctDNA analysis may enable serial assessment of changes in tumor burden (22). Moreover, ctDNA may capture genomic alterations originating from multiple tumor sites and may therefore provide information on spatial tumor heterogeneity that cannot be obtained from a single-site tissue biopsy (22–25). Evidence supporting ctDNA-based monitoring in HNSCC has increased in recent years (26–30). In patients with R/M disease, ctDNA can be detected in a substantial proportion of cases, particularly when highly sensitive tumor-informed assays are used (29, 30). Several studies have suggested that early changes in ctDNA levels during immune checkpoint inhibitor therapy are associated with subsequent clinical outcomes. Decreases or clearance of ctDNA have been associated with radiologic response and longer progression-free survival (PFS) and overall survival (OS), whereas stable or increasing ctDNA levels have generally been associated with treatment resistance and unfavorable outcomes (27–31). Molecular response patterns may also provide complementary information when early radiologic findings are inconclusive. For example, declining ctDNA levels despite apparent radiologic progression may support pseudoprogression rather than true tumor growth, although evidence for this application in HNSCC remains limited (29). Beyond treatment-response assessment, ctDNA positivity has also been shown to reflect residual or recurrent disease and may precede radiographic relapse in MRD and real-world monitoring cohorts (32–34).

Many studies evaluating tumor-derived somatic variants in HNSCC have used next-generation sequencing (NGS)-based methods, including tumor-informed assays and broader tumor-agnostic gene panels (27, 30, 32, 34–36). These approaches allow the simultaneous assessment of multiple genomic alterations and can provide extensive molecular information. Tumor-informed NGS assays may achieve high analytical sensitivity by tracking a personalized set of tumor-derived variants (34). However, their use for frequent longitudinal monitoring in routine clinical practice may be constrained by cost, turnaround time, sequencing requirements, and the need for specialized bioinformatic analysis.

Digital droplet PCR (ddPCR) offers a focused alternative for monitoring predefined tumor-specific variants. It enables rapid and highly sensitive absolute quantification using relatively small amounts of plasma-derived DNA and may be less resource-intensive than broad sequencing-based approaches, making it suitable for repeated measurements during treatment (37–39). The feasibility of ddPCR-based ctDNA quantification in plasma from patients with HNSCC has previously been demonstrated, including individualized approaches in which tumor-specific mutations are first identified by sequencing and subsequently tracked using patient-specific ddPCR assays (40). However, ddPCR can assess only a limited number of predefined alterations in each reaction. It is therefore better suited to targeted longitudinal monitoring than to comprehensive molecular profiling and should be regarded as complementary to, rather than a replacement for, NGS-based methods (40, 41). Despite these potential advantages, longitudinal ctDNA monitoring using patient-specific ddPCR assays remains comparatively underinvestigated in R/M HNSCC. Broader clinical implementation is also limited by heterogeneity in preanalytical procedures, assay design, sampling schedules, reporting thresholds, and definitions of molecular response (42–45). It therefore remains unclear whether individualized ddPCR assays can reliably capture clinically relevant ctDNA dynamics during routine systemic treatment.

In this exploratory study, we evaluated the feasibility of patient-specific ddPCR-based ctDNA monitoring in nine patients with R/M HNSCC undergoing systemic treatment and compared serial ctDNA dynamics with radiologic response and clinical course. We hypothesized that changes in ctDNA levels would reflect treatment-associated changes in disease burden and provide complementary information for longitudinal disease monitoring alongside conventional imaging.

## 2 Methods

### 2.1 Human tissue and blood samples

Comprehensive genomic profiling (CGP) was performed on tumor tissue from each patient as part of routine clinical care using FoundationOne^®^ CDx. The assay uses targeted hybrid-capture next-generation sequencing of DNA isolated from formalin-fixed, paraffin-embedded tumor tissue (46). Venous blood samples were obtained from nine patients with clinically and histopathologically confirmed head and neck cancer. Written informed consent was obtained from all patients before sample collection and subsequent molecular analyses. All study-related procedures were approved by the Cantonal Ethics Committee Zurich (BASEC-Nr. 2020-01–104 and BASEC-Nr. 2025-00988). Blood samples were collected at the Department of Medical Oncology and Hematology, University Hospital Zurich, as part of routine clinical follow-up. When available, pretreatment samples were collected before initiation of systemic therapy. Additional samples were obtained at variable, non-protocolized time points during treatment and follow-up, including at restaging visits when applicable; sampling did not follow predefined study time points. Anticancer treatment was administered according to the institutional standard of care and was not guided by the liquid biopsy results.

### 2.2 Cell-free DNA extraction

Peripheral blood samples were processed within the pre-specified handling interval. Plasma was separated by sequential centrifugation at 3,000 rpm for 10 minutes at 4 °C, followed by high-speed centrifugation at 14,000 rpm for 10 minutes at 4 °C to minimize contamination by residual cellular material. Plasma fractions were carefully transferred to fresh tubes, aliquoted, and stored at −80 °C until cfDNA extraction. Cell-free DNA was isolated from 5 mL of plasma using the MagMAX^TM^ Cell-Free Total Nucleic Acid Isolation Kit on a KingFisher^TM^ Flex Instrument according to the manufacturer’s instructions. Purified cfDNA was stored at −20 °C until downstream ddPCR analysis. cfDNA concentration was determined using the Qubit^TM^ 4 Fluorometer (Thermo Fisher Scientific, Waltham, MA, USA; cat. no. Q32866).

### 2.3 DdPCR assay design

Somatic variants detected by tissue-based genomic profiling were used to establish personalized multi-target ddPCR assays for ctDNA monitoring. Up to three single-nucleotide variants (SNVs) were selected per patient. Priority was given to known or likely oncogenic driver mutations and alterations with potential functional relevance. Where appropriate, technically suitable variants of uncertain significance were also included to increase the number of patient-specific molecular targets and potentially increase the likelihood of ctDNA detection. The selected variants were reviewed for features suggestive of germline origin, including tumor variant allele frequency and available variant annotations; variants considered potentially germline were not selected. However, because matched leukocyte DNA was not analyzed, germline origin could not be definitively established or excluded, and clonal hematopoiesis could not be assessed. Patient-specific primers and LNA-modified hydrolysis probes were designed using Beacon Designer software v8.20 (Premier Biosoft, Palo Alto, CA, USA), according to previously published ddPCR assay-development procedures (47, 48). Primer and probe sequences are provided in **Supplementary Tables 1, 2**. Primers and probes were synthesized by Integrated DNA Technologies, Inc. (IDT, Coralville, IA, USA). Allele-specific hydrolysis probes detecting the wild-type allele were labeled with hexachlorofluorescein (HEX), while probes specific for the mutant allele were labeled with 6-carboxyfluorescein (FAM) or cyanine 5 (Cy5).

### 2.4 Establishment of single-target ddPCR assays

Patient-specific digital PCR assays were established on the Naica^TM^ Crystal Digital PCR system (Stilla Technologies, Villejuif, France). Each assay was first evaluated as a single-target reaction to verify amplification efficiency, probe specificity, and allele discrimination before potential incorporation into multiplex assays. Reactions were prepared in a total volume of 25 µL using 5 µL PerfeCTa^®^ Multiplex qScript ToughMix^®^, 5X (Quantabio, Beverly, MA, USA; cat. no. 95147-250), forward and reverse primers at 1 µM each, and allele-specific hydrolysis probes at 250 nM each. Fluorescein (VWR, Radnor, PA, USA; cat. no. 0681–100 g) was added at a final concentration of 0.1 µM, and nuclease-free water (Ambion by Thermo Fisher Scientific, Waltham, MA, USA; cat. no. AM9932) was used to reach the final reaction volume. A master mix comprising all reaction components except template DNA was prepared for each run and aliquoted into individual reaction tubes. Template DNA was then added separately to each reaction.

### 2.5 Multi-target ddPCR assay establishment

Patient-specific multi-target ddPCR assays were established by combining the corresponding optimized single-target assays in a single reaction. The number of primer–probe sets included in each assay varied according to the tumor-specific variants selected for each patient. Reactions were assembled in a final volume of 25 µL using the same conditions as for the single-target ddPCR assays. Forward and reverse primers were included at final concentrations of 1 µM each, and allele-specific hydrolysis probes at 250 nM each. Depending on the number of targets included, the volume of nuclease-free water was modified accordingly to maintain the final reaction volume. All other reaction components and concentrations remained unchanged. Template DNA was added separately after preparation and aliquoting of the multi-target master mix.

### 2.6 DdPCR assay controls and analytical assessment

Synthetic double-stranded DNA fragments representing the corresponding wild-type and mutant target sequences were used for assay development and analytical assessment (gBlocks^TM^ Gene Fragments; Integrated DNA Technologies, Coralville, IA, USA). The synthetic DNA fragments had a length of 400–500 bp. During initial assay optimization, reactions containing wild-type gBlock DNA, mutant gBlock DNA, a defined mixture of wild-type and mutant gBlock DNA, and a no-template control were included. These controls were used to evaluate allele-specific signal detection, fluorescence cluster separation, and potential nonspecific amplification. GBlock stock solutions were serially diluted from an initial concentration of 10 ng/µL to a final concentration of approximately 2.5 fg/µL. Unless stated otherwise, 1 µL of the respective gBlock dilution was added per reaction, corresponding to approximately 5,000 copies/well. For analyses of patient-derived DNA, each run included a positive control containing both wild-type and mutant gBlock DNA, a wild-type control, and a no-template control. For plasma-derived cfDNA samples, a standardized DNA input volume of 9.5 µL per reaction was used. Formal assay-specific limits of blank (LoB) and detection (LoD) were not established within this exploratory pilot study. Mutant signal detectability in patient-derived samples was therefore assessed relative to the fluorescence cluster positions and background signals observed in the corresponding positive, wild-type, and no-template control reactions.

### 2.7 Digital PCR amplification and data analysis

Final reaction mixtures were loaded onto Sapphire Chips (Stilla Technologies; cat. no. C14012) according to the manufacturer’s instructions. Droplet generation and PCR amplification were performed using the Naica^TM^ Geode instrument (Stilla Technologies; cat. no. H14000). Assay-specific thermal cycling conditions are summarized in **Supplementary Table 3**. After amplification, chips were imaged on the Naica^TM^ Prism3 fluorescence reader (Stilla Technologies; cat. no. H22000) using Crystal Reader software version 3.1.6.3.SP1. Fluorescence signals were subsequently analyzed with Crystal Miner software version 3.1.6.3 (Stilla Technologies). Target concentrations were calculated automatically by the software based on Poisson statistics. Plasma samples were classified as having detectable mutant signal when mutation-specific droplets were observed within the predefined mutant fluorescence cluster and exceeded the background signal observed in the corresponding wild-type and no-template controls. In the absence of formally established assay-specific limits of blank and detection, samples without a distinguishable mutation-specific signal were reported as non-detectable under the applied assay conditions.

### 2.8 Radiologic response assessment

Imaging for treatment response assessment was performed in accordance with institutional standard of care. Imaging studies were formally interpreted by radiologists or nuclear medicine physicians and reviewed by the treating oncologist. Equivocal findings were discussed at the multidisciplinary tumor board, when appropriate. Response was classified as partial response, stable disease, progressive disease, or mixed response based on the routine clinical assessment. No study-specific response reassessment according to RECIST 1.1 or PERCIST, and no blinded central imaging review, was performed.

### 2.9 Statistical analysis

Statistical analyses were performed using GraphPad Prism version 11.0.1 (GraphPad Software, Boston, MA, USA). Continuous variables were presented as medians with interquartile ranges (IQR). Differences between two independent groups were assessed using the Mann–Whitney U test, whereas paired comparisons were analyzed using the Wilcoxon matched-pairs signed-rank test. Progression-free survival (PFS) was defined as the interval from treatment initiation to the date of first documented disease progression or death from any cause, whichever occurred first. Overall survival (OS) was defined as the interval from the date of first diagnosis of recurrent or metastatic disease to death from any cause. PFS and OS were estimated using the Kaplan–Meier method, and survival distributions between ctDNA kinetic groups were compared using the two-sided log-rank test. Patients without an event at the time of the final analysis were censored at the date of last follow-up. All tests were two-sided, and P values < 0.05 were considered statistically significant.

## 3 Results

### 3.1 Patient characteristics

A total of nine patients with clinically and histopathologically confirmed recurrent and/or metastatic head and neck squamous cell carcinoma were included in the study (**Table 1**). All patients were male. The median age at diagnosis of recurrent or metastatic disease was 65 years (interquartile range [IQR], 56–75 years; range, 49–79 years). The most common primary tumor site was the oropharynx, accounting for four patients (n = 4/9; 44.4%); including three patients with human papillomavirus (HPV)-negative tumor and one patient with HPV-positive tumor. This was followed by the nasopharynx in two patients (n = 2/9; 22.2%), including one patient with Epstein-Barr virus (EBV)-positive and one patient with EBV-negative nasopharyngeal carcinoma. The remaining patients had primary tumors of the hypopharynx, maxillary sinus, and larynx, respectively (n = 1/9 each; 11.1%). Median cumulative tobacco exposure was 30 pack-years (IQR, 5–40 pack years). Information on alcohol consumption was available for eight patients, with a median intake of 0.75 units per day (IQR, 0–2 units/day).

**Table 1:** Baseline clinicopathologic characteristics of the study cohort.

Clinicopathologic features	n = 9 (%)
Median age (years), range	65, 49-79
Sex	
Male	9 (100)
Female	0 (0)
Tumor location	
Oropharyngeal carcinoma	4 (44.4)
HPV positive (+)	1 (25)
HPV negative (-)	3 (75)
Nasopharyngeal carcinoma	2 (22.2)
EBV positive (+)	1 (50)
EBV negative (-)	1 (50)
Hypopharyngeal carcinoma	1 (11.1)
Laryngeal carcinoma	1 (11.1)
Maxillary sinus carcinoma	1 (11.1)
Smoking status (pack-years)	
Median exposure (IQR)	30 (5-40)
Alcohol consumption (units per day)	
Median intake (IQR)	0.75 (0-2)
First-line therapy	
Pembrolizumab	4 (44.4)
Carboplatin + Gemcitabine	2 (22.2)
Carboplatin + 5-Flourouracil + Pembrolizumab	1 (11.1)
Docetaxel + Cetuximab	1 (11.1)
Cisplatin + Docetaxel + 5-Fluorouracil	1 (11.1)

EBV, Epstein-Barr virus (EBV); HPV, human papillomavirus.

At study inclusion, four patients (44.4%) presented with locoregionally recurrence only, four patients (44.4%) with distant metastatic disease only, and one patient (11.1%) with both locoregional recurrence and distant metastatic disease. Prior treatment histories were heterogeneous. Four patients had previously received cisplatin-based chemoradiotherapy. One patient had received radiotherapy alone, and one patient had undergone adjuvant radiotherapy. Two patients had previously been treated with platinum–gemcitabine chemotherapy, comprising cisplatin plus gemcitabine in one patient and carboplatin plus gemcitabine in one patient. One patient had declined the recommended radiotherapy. Systemic treatment administered during ctDNA monitoring was also heterogeneous. Four patients received pembrolizumab monotherapy, and one patient received pembrolizumab in combination with carboplatin and 5-fluorouracil. The remaining patients were treated with carboplatin plus gemcitabine (22.2%), cetuximab plus docetaxel (11.1%), or cisplatin, docetaxel, and 5-fluorouracil (11.1%). Overall, five patients (55.6%) received a pembrolizumab-containing regimen. At the time of the final analysis, seven of the nine patients (77.8%) had died.

### 3.2 Baseline comprehensive genomic profiling

Baseline comprehensive genomic profiling using FoundationOne^®^ CDx was successfully performed in all nine patients. Sequencing was conducted on tissue obtained from metastatic lesions in seven patients (n = 7/9; 77.8%), including five lymph-node metastases, one brain metastasis, and one pulmonary metastasis. In the remaining two patients, tissue from the local tumor site was analyzed, comprising one nasopharyngeal tumor and one maxillary sinus tumor. The median tumor cell content of the analyzed specimens was 60% (95% CI, 40–75%; range, 20–85%). All tumors were microsatellite stable. The median tumor mutational burden was 5 Mutations/Mb (95% CI, 2–8; range, 1–10). FoundationOne^®^ CDx identified a median of 3 pathogenic or likely pathogenic genomic alterations per specimen (range, 1–12). Across all nine specimens, 49 pathogenic or likely pathogenic alterations were reported, of which 14 (28.6%) were single-nucleotide variants (SNVs), corresponding to a median of 2 SNVs per specimen (range, 0–3). No pathogenic or likely pathogenic SNV was identified in specimens from two patients. In addition, a median of 9 variants of uncertain significance (VUS) was reported per specimen (95% CI, 5-15; range, 3–16), encompassing different types of genomic alterations. The potential suitability of these variants for patient-specific ddPCR assay development was assessed according to variant type and technical assay requirements.

### 3.3 Establishment of patient-specific ddPCR assays

Based on the FoundationOne^®^ CDx results, 16 patient-specific SNVs were selected for ddPCR assay development, corresponding to a median of 2 variants per patient (range, 1–3) (**Table 2**). Nine variants (56.3%) were classified as pathogenic or likely pathogenic, whereas seven (43.8%) were classified as variants of uncertain significance. Two variants were selected in five patients, one variant in three patients, and three variants in one patient. At least one pathogenic or likely pathogenic SNV was available in five patients, whereas the selected targets in four patients consisted exclusively of SNVs classified as VUS. Overall, SNVs classified as VUS were included for five patients to increase the number of patient-specific trackable loci. *TP53* was the most frequently represented gene among the selected variants (n = 3), followed by *PIK3CA* (n = 2), while all other genes were represented by a single variant (**Table 2**). Single-target ddPCR assays were successfully established for all 16 selected SNVs, corresponding to a technical establishment rate of 100%. Following individual optimization, single-target assays were combined into patient-specific multi-target assays whenever two suitable variants were available (**Figure 1**). Owing to the three-color fluorescence detection format, each multi-target reaction was limited to two mutant targets. Both corresponding wild-type probes were labeled with HEX and detected in the same fluorescence channel, whereas the two mutation-specific probes were assigned to the two remaining fluorescence channels, allowing the two mutant alleles to be distinguished within the same reaction. For patients with only one selected variant, the respective single-target assay was retained for longitudinal monitoring. In the patient with three selected variants, the targets were distributed across two separate ddPCR reactions. All resulting patient-specific single- and multi-target reaction configurations were successfully established. In total, ten final ddPCR reaction configurations were used for the nine patients.

**Figure 1:**
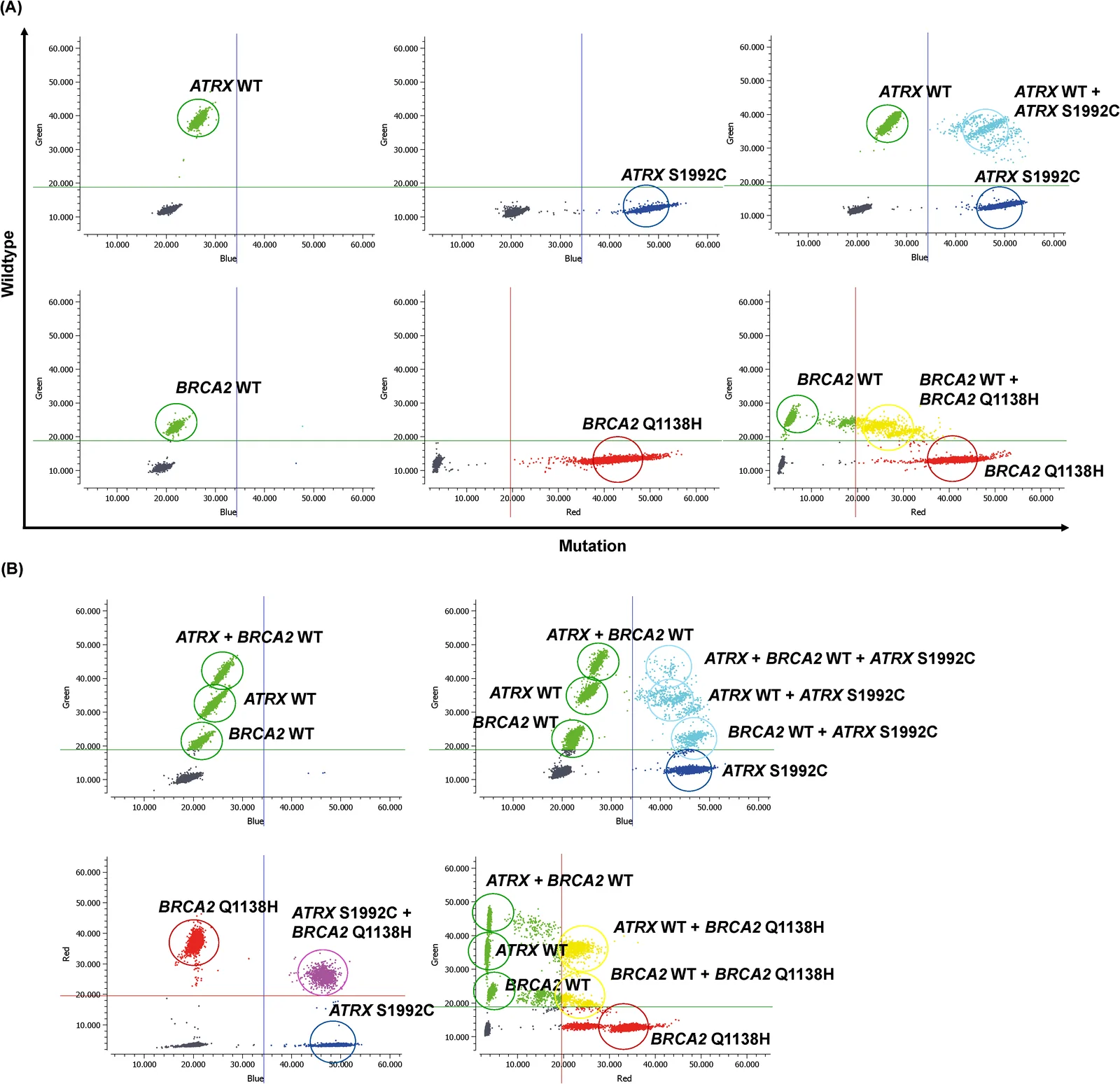
Technical establishment and optimization of patient-specific single-target and multi-target ddPCR assays. Representative assay development is shown for *ATRX* S1992C and *BRCA2* Q1138H. In-house-designed primers and allele-specific hydrolysis probes were initially evaluated in single-target reactions and optimized by adjusting the thermal cycling conditions. Where applicable, optimized single-target assays were subsequently combined into patient-specific multi-target reactions. Assay establishment was performed using synthetic double-stranded DNA fragments representing the corresponding wild-type (WT) and mutant (MUT) target sequences. **(A)** Single-target assay format. WT alleles were detected using HEX-labeled probes and are shown in green. *ATRX* S1992C-positive droplets are shown in blue, whereas droplets containing both *ATRX* WT and mutant templates form a light-blue double-positive cluster. *BRCA2* Q1138H-positive droplets are shown in red, and droplets containing both *BRCA2* WT and mutant templates form a yellow double-positive cluster. Droplets without detectable target signal are shown in gray. **(B)** Multi-target assay format. Green clusters represent droplets positive for *ATRX* WT, *BRCA2* WT, or both WT targets, with the highest green-fluorescence population corresponding to droplets containing both WT targets. Blue droplets are positive for *ATRX* S1992C, whereas red droplets are positive for *BRCA2* Q1138H. Light-blue clusters represent droplets containing *ATRX* S1992C together with *ATRX* WT, *BRCA2* WT, or both WT targets. Yellow clusters represent droplets containing *BRCA2* Q1138H together with *ATRX* WT, *BRCA2* WT, or both WT targets. Purple droplets are positive for both mutant targets, *ATRX* S1992C and *BRCA2* Q1138H. Gray droplets contain no detectable target signal. Circles highlight the principal droplet populations. Green, blue, and red threshold lines separate negative from positive droplets in the HEX, FAM, and Cy5 fluorescence channels, respectively. Cy5, cyanine 5; ddPCR, droplet digital polymerase chain reaction; FAM, 6-carboxyfluorescein; HEX, hexachlorofluorescein; MUT, mutant; WT, wild-type.

**Table 2:** Patient-specific SNVs selected for ddPCR assay development.

Patient ID	Alterations	Variant classification
P1	*BRCA1* S1383	Pathogenic
	*TP53* C238Y	Pathogenic
P2	*ATRX* S1992C	Variant of unknown significance (VUS)
	*BRCA2* Q1138H	Variant of unknown significance (VUS)
P3	*JAK1* Q231H	Variant of unknown significance (VUS)
	*MET* M619I	Variant of unknown significance (VUS)
P4	*JAK2* C427W	Variant of unknown significance (VUS)
P5	*SPEN* W1467	Likely pathogenic
P6	*PIK3CA* G122D	Pathogenic
	*TP53* I195F	Pathogenic
P7	*NFE2L2* T80A	Pathogenic
	*TP53* H179Y	Pathogenic
P8	*BARD1* T714N	Variant of unknown significance (VUS)
P9	*ATR* S1764F	Variant of unknown significance (VUS)
	*PIK3CA* E542K	Pathogenic
	*STK11* E65	Pathogenic

### 3.4 Feasibility of serial ctDNA assessment

A total of 21 plasma samples were obtained from all nine patients before and/or during systemic treatment, corresponding to a median of 2 samples per patient (range, 1–4). Serial plasma samples, defined as at least two samples per patient, were available for six of the nine patients (66.7%), whereas three patients contributed a single plasma sample. The median observation period from study inclusion to the last plasma collection was 9.0 weeks (IQR, 5.2–10.4; range, 0–26.4). Individual plasma collection dates, time points relative to treatment initiation, and sampling context are provided in **Table 3**. All 21 plasma samples were successfully processed, and technically valid ddPCR measurements were obtained for every sample (100%). Patient-specific ctDNA analysis was therefore technically feasible in all patients and across all available plasma samples. A representative example of a ctDNA-positive plasma sample is shown in **Figure 2**. Pretreatment plasma samples were available for seven of the nine patients. ctDNA was detectable before treatment initiation in three of these seven patients (42.9%), whereas no ctDNA was detected under the applied assay conditions in the remaining four patients (57.1%), despite technically valid assay performance. Among the three patients with detectable pretreatment ctDNA, two had distant metastatic disease and one had isolated locoregional recurrence. The remaining two patients were not evaluable for baseline ctDNA status because no pretreatment plasma sample was available. When all available sampling time points were considered, ctDNA was detected at least once in six of the nine patients (66.7%), including three patients with a positive sample during follow-up. In the remaining three patients (33.3%), no ctDNA was detected under the applied assay conditions throughout the available observation period. Patient-level ctDNA variant allele frequencies, percentage changes relative to baseline, and clinical outcomes, including PFS and OS, are summarized in **Table 3**. Accordingly, the inclusion of longitudinal samples increased the number of patients in whom tumor-derived variants could be detected compared with pretreatment testing alone.

**Table 3:** Patient-level plasma ctDNA variant allele frequencies and clinical outcomes.

Patient ID	Sample no.	Collection date	Time relative to treatment initiation	Sampling context	Alteration	VAF (%) sampling time	Change from baseline (%)	PFS (months)	OS (months)
P1	1	17.07.2023	Day 0 (Pre-treatment)	Baseline	*BRCA1* S1383	0.98	Reference	5 (event)	57 (censored)
P1	1	17.07.2023	Day 0 (Pre-treatment)	Baseline	*TP53* C238Y	0.00	Reference		
P1	2	07.08.2023	Day +21	Routine follow-up	*BRCA1* S1383	0.00	-100		
P1	2	07.08.2023	Day +21	Routine follow-up	*TP53* C238Y	0.00	n.c.		
P1	3	28.08.2023	Day +42	Routine follow-up	*BRCA1* S1383	0.07	-92.9		
P1	3	28.08.2023	Day +42	Routine follow-up	*TP53* C238Y	0.00	n.c.		
P2	1	14.11.2024	Day +21	Routine follow-up	*BRCA2* Q1138H	0.22	n.a.	19 (event)	59 (event)
P2	1	14.11.2024	Day +21	Routine follow-up	*ATRX* S1992C	0.00	n.a.		
P2	2	30.01.2025	Day +98	At restaging	*BRCA2* Q1138H	0.25	n.a.		
P2	2	30.01.2025	Day +98	At restaging	*ATRX* S1992C	0.00	n.a.		
P2	3	07.03.2025	Day +134	Routine follow-up	*BRCA2* Q1138H	0.13	n.a.		
P2	3	07.03.2025	Day +134	Routine follow-up	*ATRX* S1992C	0.00	n.a.		
P2	4	28.03.2025	Day +155	Routine follow-up	*BRCA2* Q1138H	0.19	n.a.		
P2	4	28.03.2025	Day +155	Routine follow-up	*ATRX* S1992C	0.00	n.a.		
P3	1	09.11.2023	Day -1 (Pre-treatment)	Baseline	*JAK1* Q231H	44.4	Reference	5 (event)	29 (censored)
P3	1	09.11.2023	Day -1 (Pre-treatment)	Baseline	*MET* M619I	0.00	Reference		
P3	2	30.11.2023	Day +20	Routine follow-up	*JAK1* Q231H	0.00	-100		
P3	2	30.11.2023	Day +20	Routine follow-up	*MET* M619I	0.00	n.c.		
P3	3	22.01.2024	Day +73	At restaging	*JAK1* Q231H	0.00	-100		
P3	3	22.01.2024	Day +73	At restaging	*MET* M619I	0.00	n.c.		
P4	1	22.06.2023	Day -7 (Pre-treatment)	Baseline	*JAK2* C427W	0.00	Reference	11 (event)	23 (event)
P4	2	29.08.2023	Day +61	Routine follow-up	*JAK2* C427W	0.00	n.c.		
P5	1	11.05.2023	Day +21	Routine follow-up	*SPEN* W1467	61.74	n.a.	3 (event)	68 (event)
P6	1	11.04.2023	Day 0 (Pre-treatment)	Baseline	*PIK3CA* G122D	0.00	Reference	2 (event)	10 (event)
P6	1	11.04.2023	Day 0 (Pre-treatment)	Baseline	*TP53* I195F	0.00	Reference		
P7	1	12.04.2023	Day -15 (Pre-treatment)	Baseline	*TP53* H179Y	0.00	Reference	1 (event)	6 (event)
P7	1	12.04.2023	Day -15 (Pre-treatment)	Baseline	*NFE2L2* T80A	0.00	Reference		
P7	2	03.05.2023	Day +6	Routine follow-up	*TP53* H179Y	0.00	n.c.		
P7	2	03.05.2023	Day +6	Routine follow-up	*NFE2L2* T80A	0.00	n.c.		
P7	3	08.06.2023	Day +42	Routine follow-up	*TP53* H179Y	0.92	newly detected		
P7	3	08.06.2023	Day +42	Routine follow-up	*NFE2L2* T80A	0.00	n.c.		
P7	4	22.06.2023	Day +56	At restaging	*TP53* H179Y	0.40	newly detected		
P7	4	22.06.2023	Day +56	At restaging	*NFE2L2* T80A	0.00	n.c.		
P8	1	13.03.2023	Day 0 (Pre-treatment)	Baseline	*BARD1* T714N	1.41	Reference	2 (event)	4 (event)
P9	1	24.03.2023	Day 0 (Pre-treatment)	Baseline	*STK11* E65	0.00	Reference	30 (event)	35 (event)
P9	1	24.03.2023	Day 0 (Pre-treatment)	Baseline	*ATR* S1764F	0.00	Reference		
P9	1	24.03.2023	Day 0 (Pre-treatment)	Baseline	*PIK3CA* E542K	0.00	Reference		
P9	2	24.04.2023	Day +31	Routine follow-up	*STK11* E65	0.00	n.c.		
P9	2	24.04.2023	Day +31	Routine follow-up	*ATR* S1764F	0.00	n.c.		
P9	2	24.04.2023	Day +31	Routine follow-up	*PIK3CA* E542K	0.00	n.c.		

Day 0 was defined as the date of initiation of the monitored systemic treatment. Samples collected before the first treatment dose on that date are labeled “Day 0 (pre-treatment)”; samples collected before that date are reported as negative days. VAF values of 0.00% indicate that no mutant signal was detected while the wild-type/reference signal was measured. Percentage change from baseline was calculated as [(follow-up VAF − baseline VAF)/baseline VAF] × 100. Percentage changes are calculated from the observed VAF values and do not imply absolute biological absence of the variant. ctDNA, circulating-tumor DNA; n.a., not available; n.c., not calculable; VAF, variant allele frequency.

**Figure 2:**
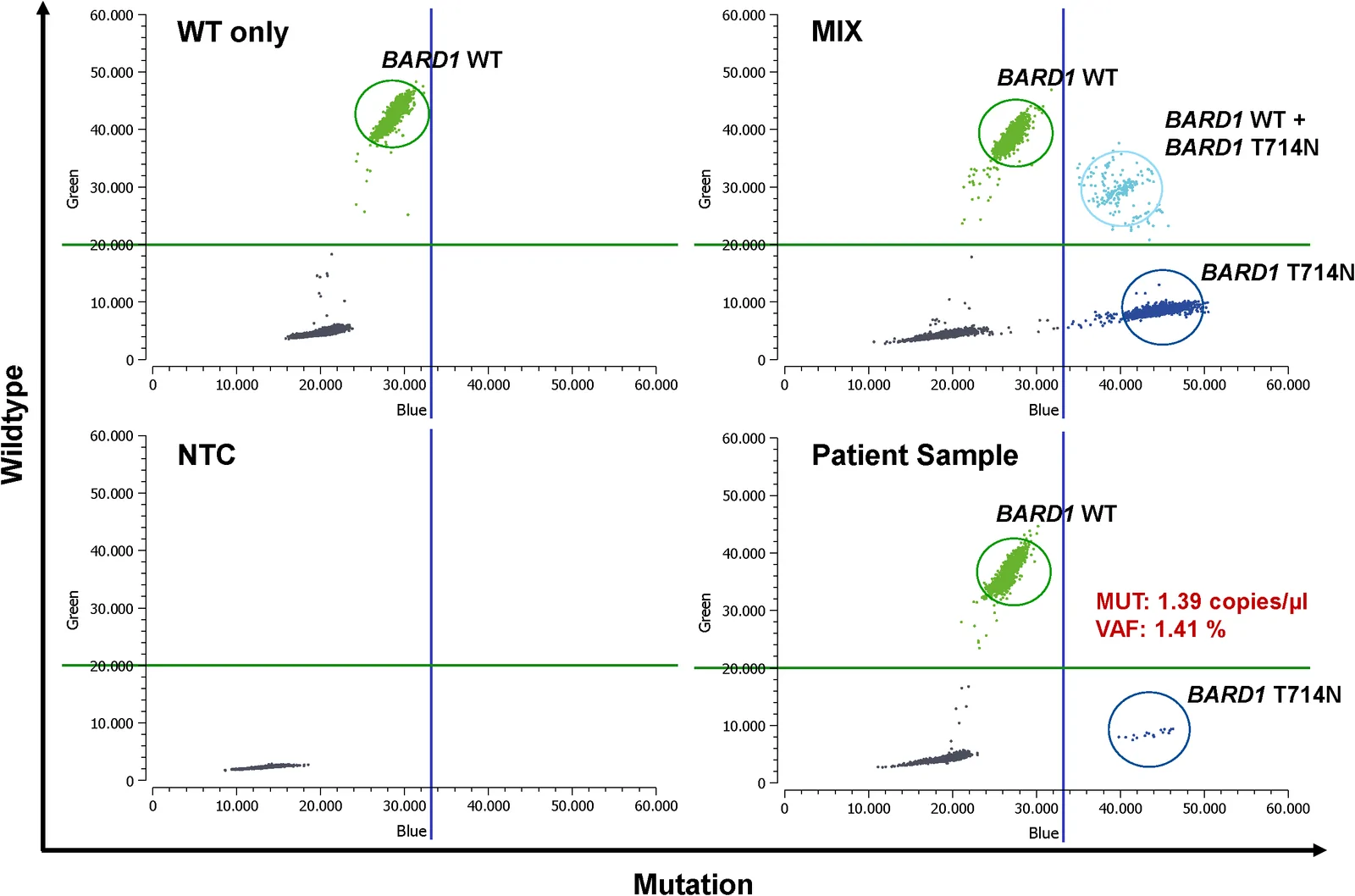
Representative detection of *BARD1* T714N in patient-derived plasma cfDNA using a patient-specific ddPCR assay. Representative two-dimensional fluorescence plots are shown for a wild-type-only control (WT only), a defined mixture of wild-type and mutant synthetic DNA (MIX), a no-template control (NTC), and a patient-derived plasma cfDNA sample. The *BARD1* wild-type allele was detected using a HEX-labeled probe, whereas the *BARD1* T714N mutant allele was detected using a FAM-labeled probe. WT-positive droplets are shown in green, *BARD1* T714N-positive droplets in blue, and droplets containing both WT and mutant target molecules form a light-blue double-positive cluster. Droplets without detectable target signal are shown in gray. In the patient sample, the *BARD1* T714N mutant concentration was 1.39 copies/µL, corresponding to a variant allele fraction of 1.41%. Circles highlight the principal positive droplet populations. The green horizontal threshold line separates HEX-negative from HEX-positive droplets, whereas the blue vertical threshold line separates FAM-negative from FAM-positive droplets. Droplet clusters and thresholds were assigned using Crystal Miner software. cfDNA, cell-free DNA; ddPCR, droplet digital polymerase chain reaction; FAM, 6-carboxyfluorescein; HEX, hexachlorofluorescein; MIX, wild-type/mutant mixture; MUT, mutant; NTC, no-template control; VAF, variant allele fraction; WT, wild-type.

### 3.5 Association of ctDNA kinetics with clinical and radiologic outcomes

At first restaging, eight of nine patients underwent FDG PET/CT, whereas one patient underwent CT of the neck, thorax, and abdomen. The median interval from treatment initiation to first restaging was 66 days (IQR, 57–100; range, 53–119). Among the six patients with serial plasma sampling, ctDNA kinetics were concordant with radiologic treatment assessment in five patients (83.3%). The single patient with increasing ctDNA levels developed radiologically confirmed progressive disease. Among the five patients with decreasing or stable ctDNA levels, two achieved a partial response and two had stable disease on imaging. The remaining patient showed a mixed radiologic response and represented the only discordant case between molecular and imaging-based assessment (**Table 3**). A representative example of concordant molecular and radiologic response is shown in **Figure 3**. In this patient, the plasma concentration of the tumor-specific *BRCA1* S1383* variant decreased from 0.48 copies/µL at pretreatment baseline to undetectable levels during capecitabine treatment and remained markedly below baseline at the subsequent assessment. This molecular decline was accompanied by a pronounced reduction in circulating EBV-DNA and a radiologically confirmed partial response at three months. In an exploratory Kaplan–Meier analysis, PFS and OS appeared shorter in the single patient with increasing ctDNA than in the five patients with decreasing or stable ctDNA levels (nominal log-rank *P* = 0.0253 for both comparisons; **Supplementary Figure 1**). Given the highly unbalanced group sizes, including only one patient in the ctDNA-increase group, these *P* values should be interpreted cautiously and do not establish a prognostic association. A reliable Cox proportional hazards estimate could not be obtained because of complete separation of the survival outcomes.

**Figure 3:**
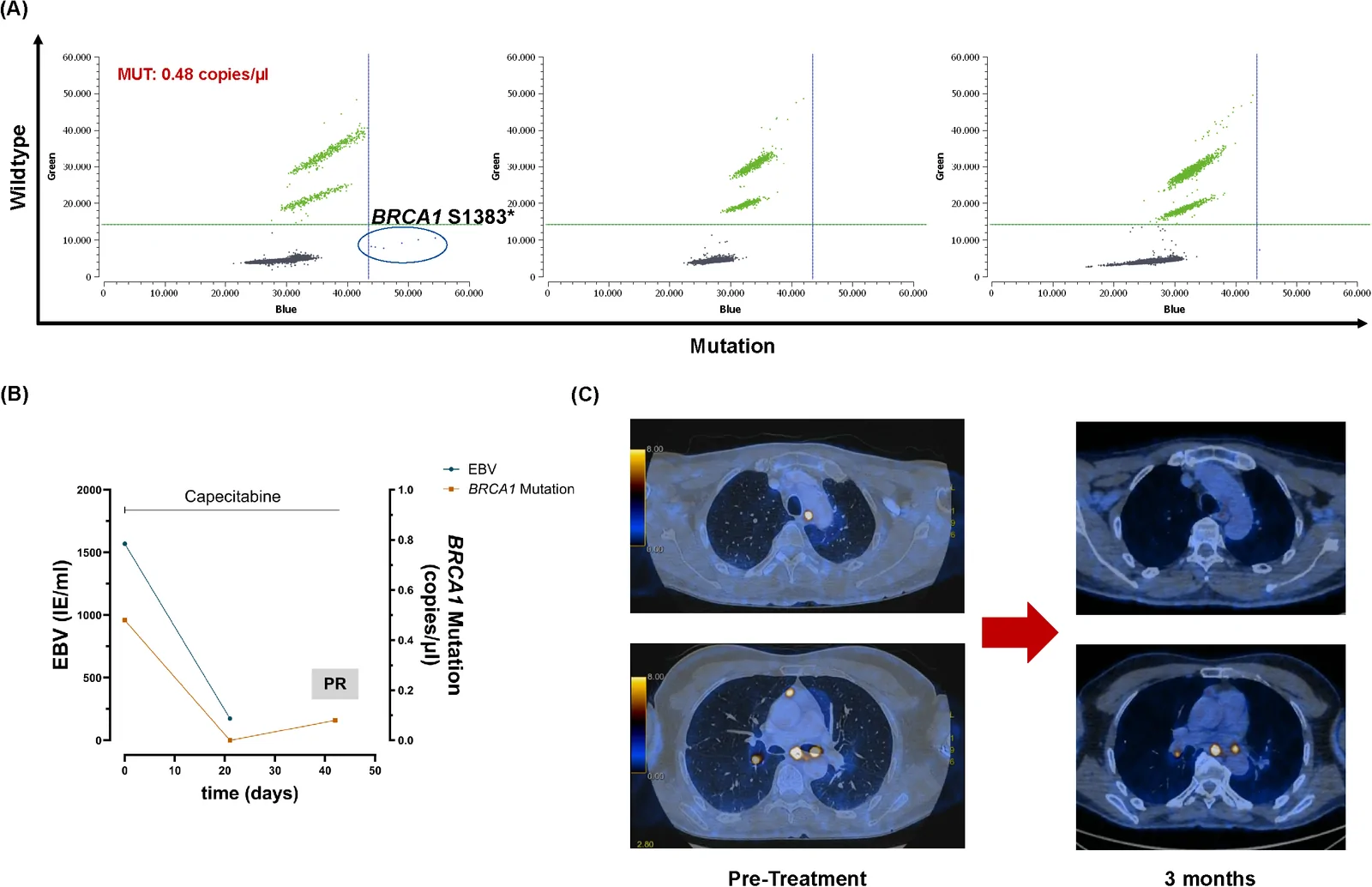
Concordance between plasma cfDNA kinetics and radiologic treatment response. Representative longitudinal monitoring of the tumor-specific *BRCA1* S1383* variant in plasma cfDNA, circulating Epstein–Barr virus (EBV) DNA, and radiologic treatment response in a patient receiving capecitabine. **(A)** Representative two-dimensional ddPCR fluorescence plots from serial plasma samples. *BRCA1* WT positive droplets are shown in green, *BRCA1* S1383*-positive droplets in blue, and droplets without detectable target signal in gray. The circle highlights the mutation-positive droplet population. The green horizontal and blue vertical threshold lines separate negative from positive droplets in the HEX and FAM fluorescence channels, respectively. **(B)** Longitudinal changes in circulating EBV DNA and plasma *BRCA1* S1383* concentration during capecitabine treatment. PR denotes the radiologically assessed partial response. **(C)** Representative axial FDG PET/CT images obtained before treatment and three months after treatment initiation. cfDNA, cell-free DNA; ddPCR, droplet digital polymerase chain reaction; EBV, Epstein–Barr virus; FAM, 6-carboxyfluorescein; HEX, hexachlorofluorescein; MUT, mutant; FDG PET/CT, Fluordeoxyglucose positron emission tomography/computed tomography; PR, partial response; WT, wild-type.

## 4 Discussion

In this pilot study, we evaluated the feasibility of patient-specific ddPCR-based ctDNA monitoring in patients with recurrent and/or metastatic HNSCC. Hereby, patient-specific assays could be technically established for all selected SNVs, all available plasma samples could be successfully analyzed, and ctDNA was detectable at least once in two-thirds of the patients. Among the six patients with serial sampling, ctDNA kinetics showed descriptive agreement with radiologic treatment assessment in five cases. Given the small number of patients, this observation should be interpreted cautiously. These findings provide proof of principle that variants identified through routine tissue genomic profiling can be translated into individualized assays for longitudinal plasma monitoring.

Our findings are consistent with a growing body of evidence indicating that longitudinal ctDNA kinetics may be more clinically informative than a single pretreatment measurement. In a cohort of 53 patients with R/M HNSCC receiving chemotherapy or immune checkpoint blockade, Taylor et al. found that baseline ctDNA abundance was not associated with PFS or OS, whereas changes in ctDNA variant allele frequency after one treatment cycle were associated with both outcomes (29). Notably, declining ctDNA also identified patients with more favorable survival despite apparent early radiologic progression, suggesting that molecular kinetics may provide complementary information in clinically or radiologically ambiguous situations (29). Similarly, Honoré et al. reported 74% concordance between early ctDNA kinetics and imaging response in patients receiving PD-1 inhibition (27). More recently, Ruiz-Torres et al. analyzed 137 samples from 16 patients receiving immune checkpoint blockade and found that on-treatment ctDNA negativity was associated with disease control, PFS, and OS, whereas early ctDNA increases were associated with progression (30). The observed agreement in five of six patients is numerically in line with these reports; however, the small number of evaluable patients precludes a reliable comparison.

Pretreatment ctDNA was detectable in three of seven evaluable patients, corresponding to a detection rate of 42.9%. This rate was lower than those reported by Honoré et al. in comparable R/M HNSCC cohorts: 35 of 44 (80%) using a tumor-agnostic assay and 25 of 29 patients (86%) using a tumor-informed assay (27, 28). Lower tumor burden may have contributed to the difference, as our cohort included patients with locoregional-only recurrence. However, tumor volumes were not quantified, so we cannot determine whether tumor burden was the main explanation. Differences in assay design and analytical sensitivity may also have contributed: our assays tracked one to three predefined SNVs per patient, and formal assay-specific LoB and LoD were not established. These differences limit direct comparison of detection rates across studies. Importantly, ctDNA was first detected during follow-up in three additional patients. Two of these patients had no pretreatment plasma sample available; in one patient, ctDNA was detected during follow-up after a negative pretreatment result. Thus, serial sampling identified ctDNA in additional patients, although the absence of pretreatment samples in two patients limits interpretation of when ctDNA first became detectable. A strength of the present approach was the successful establishment of assays for all selected variants. Individualized digital PCR strategies have previously been used in HNSCC, including tumor sequencing followed by patient-specific tracking of selected mutations. Kogo et al. demonstrated the feasibility of such an approach for longitudinal disease monitoring, although suitable variants and technically functional assays were not available for every screened patient (40). In our study, technically suitable SNVs classified as VUS were included when too few pathogenic or likely pathogenic SNVs were available, enabling at least one target to be selected for every patient. Our cohort was substantially smaller than those in recent studies of tumor-agnostic (27) and tumor-informed (28) assays. Accordingly, our findings should be interpreted as feasibility data rather than comparative evidence. All 16 selected SNVs were successfully established as ddPCR assays, and technically valid results were obtained for all 21 plasma samples. The potential added value of this strategy lies in translating variants identified through routine tissue profiling into focused assays for serial plasma monitoring; this study was not designed to demonstrate superior performance over broader sequencing-based approaches. As assay costs, turnaround time, and resource use were not directly assessed, cost-effectiveness remains to be established in prospective comparative studies. For monitoring purposes, the functional relevance of a variant may be less important than its tumor specificity, analytical performance, and persistence over time. Nevertheless, VUS may be subclonal or absent from some disease sites. Because matched leukocyte sequencing was not performed, germline origin and clonal hematopoiesis could not be definitively excluded for the tracked variants, including TP53. Future workflows should therefore preferentially select clonal tumor-specific variants and incorporate matched normal sequencing where feasible. Known cancer-associated driver mutations identified in tumor tissue may provide biologically relevant targets for patient-specific ctDNA monitoring. Their usefulness depends, however, on whether they are sufficiently represented across the patient’s tumor sites and detectable in plasma; intratumoral heterogeneity may limit the representativeness of a single tracked variant (23, 25). Patient-specific panels can track alterations identified in an individual tumor, but require prior tissue profiling and assay development and may miss subclonal or newly emerging variants (28). Predefined tumor-agnostic multigene panels can be applied without patient-specific assay design, but are restricted to the loci they cover (27). Because we did not compare these approaches head to head, our findings demonstrate the technical feasibility of patient-specific ddPCR but do not establish superior sensitivity or clinical performance. The focused nature of ddPCR represents both an advantage and a limitation. Once established, ddPCR permits rapid quantitative assessment of predefined variants using limited plasma input and without repeated broad sequencing. However, it cannot identify newly emerging alterations or fully capture spatial and temporal tumor heterogeneity. Monitoring more than one variant may reduce the risk that an individual subclonal marker becomes uninformative, but the three-color format used in this study limited each reaction to two mutant targets. ddPCR should therefore be considered a focused monitoring tool that complements rather than replaces comprehensive genomic profiling. The only discordant patient in our cohort had decreasing or stable ctDNA despite a mixed radiologic response. This may reflect different responses across disease sites, with the plasma signal representing an aggregate of responding and progressing lesions, or the tracked variants may not have represented the progressing clone.

The exploratory survival analysis showed shorter observed PFS and OS in the single patient with increasing ctDNA than in the five patients with decreasing or stable ctDNA levels. The nominal log-rank *P* values were 0.0253 for both comparisons; however, these results were driven by a single patient and do not establish a prognostic association. Complete separation also prevented reliable estimation of a Cox hazard ratio. Furthermore, ctDNA kinetic groups were defined only after serial sampling, whereas survival was calculated from the diagnosis of recurrent or metastatic disease. This time-dependent classification may introduce bias. The survival findings should therefore be considered hypothesis-generating. Larger studies should use predefined early sampling windows and landmark analyses or time-dependent models to assess the prognostic relevance of ctDNA kinetics.

Several limitations should be acknowledged. This was a small, single-center study of nine male patients with heterogeneous primary tumor sites, recurrence patterns, previous treatments, and systemic treatment regimens. Only six patients provided serial samples, two lacked pretreatment samples, and sampling schedules were not standardized. The small number of patients with serial samples and the variable sampling intervals limited our ability to characterize ctDNA trajectories consistently, identify an optimal sampling window, or assess the robustness of the observed associations. Accordingly, our results represent an exploratory assessment of serial ctDNA measurements rather than a formal longitudinal analysis, and the observed agreement with radiologic assessment and the survival comparisons should be interpreted as hypothesis-generating. Formal assay-specific limits of blank and detection were not established; consequently, negative findings indicate non-detectability under the applied assay conditions rather than confirmed absence of ctDNA. The restricted number of tracked loci may also have limited detection in patients with low-volume or predominantly locoregional disease.

In conclusion, individualized ddPCR-based ctDNA monitoring was technically feasible and showed descriptive agreement with radiologic treatment assessment in five of six evaluable patients with R/M HNSCC. Serial sampling identified ctDNA in additional patients beyond pretreatment assessment alone, emphasizing the potential value of longitudinal monitoring. Although the clinical associations remain exploratory, these findings support prospective evaluation in larger cohorts using standardized sampling schedules, formal analytical validation, and predefined molecular response criteria.

## Data Availability

The raw data supporting the conclusions of this article will be made available by the authors, without undue reservation.
