# Supplementary material for: Sequencing-informed, patient-specific droplet digital PCR assays for circulating tumor DNA monitoring in recurrent or metastatic head and neck cancer: a pilot study
Source: Front Oncol. 2026 Oct 30;16:1984504. doi: 10.3389/fonc.2026.1984504 (PMC13759421; doi:10.3389/fonc.2026.1984504)
Supplement: Supplementary file 1 [file Table1.docx]

Supplementary Material

# 1 Supplementary Figures and Tables

## Supplementary Figure 1 - Exploratory survival analysis and longitudinal ctDNA sampling.

Kaplan–Meier estimates of (A) progression-free survival (PFS) and (B) overall survival (OS) in patients with serial plasma sampling, stratified by increasing ctDNA levels (n = 1) versus decreasing or stable ctDNA levels (n = 5). (C) Individual plasma sampling time points relative to treatment initiation for the six patients with serial sampling. Each dot represents one sample; orange and teal indicate increasing and decreasing/stable ctDNA levels, respectively. PFS was calculated from initiation of systemic treatment until radiologic or clinical progression or death, whereas OS was calculated from the diagnosis of recurrent or metastatic disease until death or last follow-up. Survival distributions were compared using the two-sided log-rank test; the resulting *P* values are exploratory and should be interpreted cautiously given the small and highly unbalanced groups. Vertical tick marks indicate censored observations, and the numbers at risk are shown below panels A and B. ctDNA, circulating tumor DNA; OS, overall survival; PFS, progression-free survival.

## Supplementary Table 1 – ddPCR Primer Sequences

| Gene | Target | Direction | Sequence |
| --- | --- | --- | --- |
| *ATR* | S1764 | forward | 5’ – GTA CGT GTT TAA TTC ATC TG – 3’ |
| *ATR* | S1764 | reverse | 5’ – CTC TGA ATG TCA CCT TAA – 3’ |
| *ATRX* | S1992C | forward | 5’ – CCT ACT TAT GTT CCA TTT GTC – 3’ |
| *ATRX* | S1992C | reverse | 5’ – CAG CAT CAG CAT CTG TAA – 3’ |
| *BARD1* | T714N | forward | 5’ – CAT GGT ATG CGA CTG TAT TG – 3’ |
| *BARD1* | T714N | reverse | 5’ – GCC AGA TCC TCA GTA GAA – 3’ |
| *BRCA1* | S1383 | forward | 5’ – CCT GAG TGG TTA AAA TGT C – 3’ |
| *BRCA1* | S1383 | reverse | 5’ – GTG TGA GAG TGA AAC AAG – 3’ |
| *BRCA2* | Q1138H | forward | 5’ – TCA GGA AGT CAG TTT GAA – 3’ |
| *BRCA2* | Q1138H | reverse | 5’ – GGT CTT TAA GAT AGT CAT CTG – 3’ |
| *JAK1* | Q231H | forward | 5’ – CTG TCA CAA ATG GTC TTG – 3’ |
| *JAK1* | Q231H | reverse | 5’ – TCC AGA AAC ATT GAA TAA GTC – 3’ |
| *JAK2* | C427W | forward | 5’ – CAG GTA ATC AGA CTG GAC – 3’ |
| *JaK2* | C427W | reverse | 5’ – ACC ACT AAT AAG TGT GAC ATA – 3’ |
| *MET* | M619I | forward | 5’ – CTC CTT GGA AAT GAG AGC – 3’ |
| *MET* | M619I | reverse | 5’ – CCC AGC AAA GCA TTT TAA – 3’ |
| *NFE2L2* | T80A | forward | 5’ – TGG CTG AAT TGG GAG AAA – 3’ |
| *NFE2L2* | T80A | reverse | 5’ – AGC GAC GGA AAG AGT ATG -3’ |
| *PIK3CA* | E542K | forward | 5’ – GCT CAA AGC AAT TTC TAC A – 3’ |
| *PIK3CA* | E542K | reverse | 5’ – ACC TGT GAC TCC ATA GAA – 3’ |
| *PIK3CA* | G122D | forward | 5’ – GCT GTG TAT GTA ATA GAA TGT TA - 3’ |
| *PIK3CA* | G122D | reverse | 5’ – GTC CTG TAC TTC TGG ATC – 3’ |
| *STK11* | E65 | forward | 5’ – ATC GGC AAG TAC CTG ATG – 3’ |
| *STK11* | E65 | reverse | 5’ – CCT TCG CAA CTT CTT CTT C – 3’ |
| *SPEN* | W1467 | forward | 5’ – GAG CTA AAT CCC TCT CTT C – 3’ |
| *SPEN* | W1467 | reverse | 5’ – CAA CCT TTT CTT TAT CTT TGT TG -3’ |
| *TP53* | C238Y | forward | 5’ – CAG TGT GAT GAT GGT GAG – 3’ |
| *TP53* | C238Y | reverse | 5’ – CTG TGT TAT CTC CTA GGT TG – 3’ |
| *TP53* | H179Y | forward | 5’ – CTC ACC ATC GCT ATC TGA – 3’ |
| *TP53* | H179Y | reverse | 5’ – CCA TCT ACA AGC AGT CAC – 3’ |
| *TP53* | I195F | forward | 5’ – CTG TCA TCC AAA TAC TCC A – 3’ |
| *TP53* | I195F | reverse | 5’- CCT CAC TGA TTG CTC TTA – 3’ |

## Supplementary Table 2 – ddPCR Probe Sequences

| Gene | Target | Direction | Sequence |
| --- | --- | --- | --- |
| *ATR* | S1764 | Wildtype | 5’-/5HEX/CCA C+TC G+GA C+CT A+TT AA/3IABkFQ/-3’ |
| *ATR* | S1764 | Mutant | 5’-/56-FAM/CCA C+CT G+AA C+CT A+TT AA/3IABkFQ/-3’ |
| *ATRX* | S1992C | Wildtype | 5’-/5HEX/TCT T+CT T+CT A+AT C+CA ATC/3IABkFQ/-3’ |
| *ATRX* | S1992C | Mutant | 5’-/56-FAM/TCT T+CT T+GT A+AT C+CA AGC/3IABkFQ/-3’ |
| *BARD1* | T714N | Wildtype | 5’-/5HEX/TGGTC+TGA+GTC+ACG+TCAC/3IABkFQ/-3’ |
| *BARD1* | T714N | Mutant | 5’-/56-FAM/TGG TC+T GA+T TC+A CG+T CAC/3IABkFQ/-3’ |
| *BRCA1* | S1383 | Wildtype | 5’-/5HEX/TAG C+CC T+GA C+CA G+TC TT/3IABkFQ/-3’ |
| *BRCA1* | S1383 | Mutant | 5’-/56-FAM/TAG C+CC T+CA G+CA G+TC TT/3IABkFQ/-3’ |
| *BRCA2* | Q1138H | Wildtype | 5’-/5HEX/TAC T+CT T+CT G+CA A+TA TGT/3IABkFQ/-3’ |
| *BRCA2* | Q1138H | Mutant | 5’-/5Cy5/ TAC T+CT T+GT G+CA A+TA TGT/3IABkFQ/-3’ |
| *JAK1* | Q231H | Wildtype | 5’-/5HEX/ ATC AG+A CA+G AG+G AA+C CTT/3IABkFQ/ -3’ |
| *JAK1* | Q231H | Mutant | 5’-/56-FAM/ ATC AG+A CA+C AG+G AA+C CTT/3IABkFQ/ -3’ |
| *JAK2* | C427W | Wildtype | 5’-/5HEX/ TTC G+AT G+CA G+TC C+TA AG/3IABkFQ/ -3’ |
| *JaK2* | C427W | Mutant | 5’-/56-FAM/ TTC G+AT G+GA G+TC C+TA AG/3IABkFQ/ -3’ |
| *MET* | M619I | Wildtype | 5’-/5HEX/ AGA G+CA C+GA T+GA A+TA CGT/3IABkFQ/ -3’ |
| *MET* | M619I | Mutant | 5’-/5Cy5/ AGA G+CA C+GA T+AA A+TA CGT/3IABkFQ/ -3’ |
| *NFE2L2* | T80A | Wildtype | 5’-/5HEX/TCA C+CT G+TC T+CT T+CA TC/3IABkFQ/-3’ |
| *NFE2L2* | T80A | Mutant | 5’-/56-FAM/TCA C+CT G+CC T+CT T+CA TC/3IABkFQ/-3’ |
| *PIK3CA* | E542K | Wildtype | 5’-/5HEX/CCT+CTC+TCT+GAA+ATC ACT G/3IABkFQ/-3’ |
| *PIK3CA* | E542K | Mutant | 5’-/5Cy5/CCT+CTC+TCT+AAA+ATC ACT G/3IABRQSp/-3’ |
| *PIK3CA* | G122D | Wildtype | 5’-/5HEX/TTT G+CT A+TC G+GC ATG C/3IABkFQ/-3’ |
| *PIK3CA* | G122D | Mutant | 5’-/5Cy5/TTT G+CT A+TC G+AC ATG C/3IAbRQSp/-3’ |
| *STK11* | E65 | Wildtype | 5’-/5HEX/AAG GTC + AAG +GAG + GTG CTG/3IABkFQ/-3’ |
| *STK11* | E65 | Mutant | 5’-/5Cy5/AAG GTG+AAG+TAG+GTG CTG/3IAbRQSp/-3’ |
| *SPEN* | W1467 | Wildtype | 5’-/5HEX/CGG GA+G TC+C CA+A TC+A AGA/3IABkFQ/-3’ |
| *SPEN* | W1467 | Mutant | 5’-/56-FAM/CGG GA+G TC+T CA+A TC+A AGA/3IABkFQ/-3’ |
| *TP53* | C238Y | Wildtype | 5’-/5HEX/ACA+TGT+GTA+ACA+GTTCCT/3IABkFQ/-3’ |
| *TP53* | C238Y | Mutant | 5’-/5Cy5/ACA+TGT+ATA+ACA+GTTCCT/3IABkFQ/-3’ |
| *TP53* | H179Y | Wildtype | 5’-/5HEX/CCC+CAC+CAT+GAG CGC/3IABkFQ/ -3’ |
| *TP53* | H179Y | Mutant | 5’-/5Cy5/CCC+CAC+TAT+GAG CGC/3IAbRQSp/-3’ |
| *TP53* | I195F | Wildtype | 5’-/5HEX/TCC A+CT C+GG A+TA A+GA TGC/3IABkFQ/-3’ |
| *TP53* | I195F | Mutant | 5’-/56-FAM/TCC A+CT C+GG A+AA A+GA TGC/3IABkFQ/-3’ |

Cy5, cyanine 5; FAM, 6-Carboxyflourescein; HEX, Hexachloro-Flouresceine; 3IABkFQ, 3’ Iowa Black® FQ, Quencher from IDT

## Supplementary Table 3 – ddPCR Conditions

| **Parameter** | **Setting** |
| --- | --- |
| **Droplet Generation** | |
| Partitioning temperature | 40°C «Sapphire V1» |
| **Thermal cycling** | |
| Initial denaturation / polymerase activation | 95.0°C for 10 min |
| Number of cycles | 45 |
| Denaturation | 95.0°C for 30 s |
| Annealing / extension | 58.0°C ± 0.5°C for 30 s |
| Cycle closure | Close Cycle |
| Droplet preservation | Release P, Sapphire V1 |
| **Readout** | |
| Reader | Crystal Reader |
| Exposure time | FAM: 75 ms; HEX: 150 ms; Cy5: 50 ms |
